# Low-dose versus standard-dose intravenous immunoglobulin in generalized myasthenia gravis: a prospective single-center cohort study

**DOI:** 10.3389/fneur.2026.1780647

**Published:** 2026-04-28

**Authors:** Haocheng Luo, Li Dong, Li Yang, Yufei Deng, Chaoyue Zhang, Xuxiang Zhang, Liqing Hu, Zhenyu Wu, Xiaojun Yang, Qilong Jiang

**Affiliations:** 1 The First Clinical Medical College of Guangzhou University of Chinese Medicine, Guangzhou, China; 2 The First Affiliated Hospital of Chinese Medicine, Guangzhou University of Chinese Medicine, Guangzhou, China

**Keywords:** dose comparison, intravenous immunoglobulin, MG-ADL, myasthenia gravis, prednisone, prospective cohort, QMG

## Abstract

**Background:**

Generalized myasthenia gravis (gMG) is commonly treated with high-dose intravenous immunoglobulin (IVIG), typically at cumulative doses near 2 g/kg, during clinical worsening. However, the minimal effective IVIG exposure remains uncertain.

**Methods:**

In this prospective single-center cohort study, we enrolled 34 adults with AChR-positive gMG who received either low-dose IVIG (target ≤0.2 g/kg/day; *n* = 21) or standard-dose IVIG (target 0.4 g/kg/day; *n* = 13) according to routine clinical practice. Clinical outcomes were assessed using the Myasthenia Gravis Activities of Daily Living (MG-ADL) scale and the Quantitative Myasthenia Gravis (QMG) score at baseline and at weeks 1, 2, 4, 8, and 12. For the index IVIG course, infusion days, cumulative dose, and primary treatment indication were recorded. Oral prednisone use was evaluated as a short-term steroid-sparing outcome. Between-group comparisons were explored using regression models adjusted for age and thymoma status.

**Results:**

Both regimens were associated with clinically meaningful improvement in MG-ADL and QMG through week 12, and no statistically significant between-group differences were detected at any follow-up visit. Most patients received 5 infusion days. After weight-based conversion, the low-dose group clustered around a cumulative exposure of approximately 1.0 g/kg, whereas the standard-dose group clustered around approximately 2.0 g/kg. Acute exacerbation was the most common indication in both groups, whereas maintenance therapy was more frequent in the standard-dose cohort. Prednisone requirements did not decline over 12 weeks in either group.

**Conclusion:**

In this prospective observational cohort, low-dose IVIG was associated with short-term clinical improvement comparable to that observed with standard-dose IVIG, without a clear steroid-sparing signal. These findings should be interpreted cautiously given the small sample size, baseline imbalance, and non-randomized design.

## Introduction

Myasthenia gravis (MG) is an autoimmune neuromuscular disorder characterised by immunoglobulin G antibodies targeting the acetylcholine receptor or associated postsynaptic proteins. The resulting immune complex formation triggers complement-mediated damage, causing fluctuating, fatigable weakness that can become life-threatening (1). Symptomatic interventions such as acetylcholinesterase inhibitors and thymectomy provide relief but do not control the underlying immune attack; patients may therefore require rapid immunomodulation during exacerbations to prevent respiratory compromise.

Standard rescue options for worsening MG include plasma exchange and intravenous immunoglobulin (IVIG) (1). Novel biologics that inhibit complement or the neonatal Fc receptor have recently entered clinical practice (2). Although these targeted agents offer new avenues for disease control, their high cost and restricted availability underscore the continuing need to optimise established treatments.

**Figure 1:**
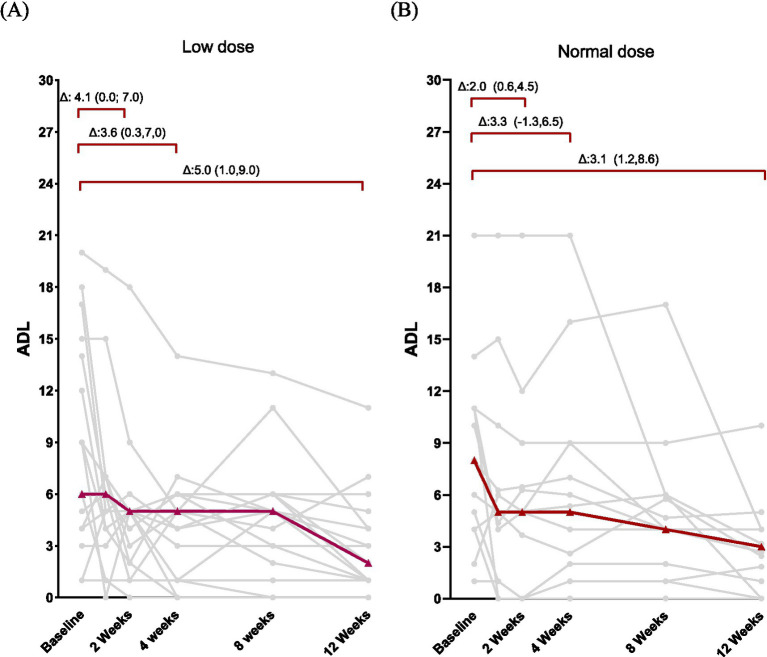
**(A,B)** Changes in myasthenia gravis activities of daily living (MG-ADL) scores for patients treated low-dose or standard-dose human immunoglobulin (baseline, weeks 1, 2, 4, 8, and 12). Individual trajectories (gray) and median changes from baseline with interquartile ranges (red) are shown (Supplementary Figures 1C,D). Quantitative myasthenia gravis (QMG) scores in individual patients treated with low-dose or standard-dose human immunoglobulin (baseline, weeks 1, 2, 4, 8, and 12) (Supplementary Figure 1E). Distributions of minimally improved MG-ADL scores from baseline to visit 5 (week 12) for low-dose vs. standard-dose immunoglobulin cohorts (Supplementary Figure 1F). Distributions of minimally improved QMG scores from baseline to visit 5 (week 12) for both dosing regimens. AChR-Ab = acetylcholine receptor antibody; gMG = generalized myasthenia gravis (Supplementary Figure 1G). Distribution of prednisone dose at V0 and V5 by group (box = IQR with median; whiskers = 1.5 × IQR; dots = individual patients) (Supplementary Figure 1H). Mean change in prednisone dose from V0 to V5 for each group; arrows annotate the magnitude of change (mg). Y-axis starts at 0 (Supplementary Figure 1I). Individual paired trajectories from V0 to V5 with overlaid group means (thick lines). Data presentation in the main tables follows median [IQR]; statistical tests used for within-group changes were paired *t*-test when the distribution of paired differences was normal, otherwise Wilcoxon signed-rank test; between-group differences were assessed using independent-samples *t*-test (equal variance), Welch’s *t*-test (unequal variance), or Mann–Whitney U test according to data distribution (Supplementary Figure 1J). Observed longitudinal MG-ADL and QMG trajectories by treatment group over 12 weeks. Panel J-A shows MG-ADL scores and panel J-B shows QMG scores for the low-dose and standard-dose IVIG groups. Points and lines indicate observed group means, and shaded bands represent 95% confidence intervals. Assessments were performed at baseline and at weeks 1, 2, 4, 8, and 12 (Supplementary Figure 1K). Index IVIG course: exposure metrics and primary treatment indication. Panel K-A shows the calculated daily IVIG dose (g/kg/day), panel K-B the calculated cumulative IVIG dose (g/kg), panel K-C infusion days, and panel K-D the within-group distribution of the primary indication for IVIG. Weight-adjusted dose metrics were derived by combining gram amounts recorded in the revision supplement sheet with baseline body weight from the primary dataset.

IVIG is a pooled IgG preparation that modulates immunity by saturating the neonatal Fc receptor, neutralising pathogenic antibodies and inhibiting complement activation (3, 4). These mechanisms are saturable; once Fc receptors and complement components are engaged, additional immunoglobulin may not confer extra benefit. Nonetheless, consensus guidelines continue to recommend cumulative doses of approximately 2 g kg^−1^ for MG exacerbations based largely on historical precedent (see Figure 1).

Limited evidence suggests lower doses may suffice. A small trial reported equivalent clinical improvement with 1 g kg^−1^ and 2 g kg^−1^ IVIG (5), and pharmacodynamic considerations imply a threshold effect (3). No prospective study has directly compared half-daily dosing with the conventional schedule or examined whether dose reduction permits corticosteroid tapering. Moreover, IVIG is expensive and frequently scarce; cost analyses estimate that dose titration could yield substantial savings and improve access (6). Determining the minimal effective dose is therefore a clinically and economically relevant goal.

To address this gap, we conducted a prospective single-center cohort study comparing a low-dose IVIG regimen (target ≤0.2 g kg^−1^ d^−1^) with the conventional 0.4 g kg^−1^ d^−1^ regimen in adults with generalized MG. Our primary aim was to compare short-term clinical outcomes over 12 weeks using MG-ADL and QMG. We also characterized the actual exposure of the index IVIG course, including infusion days, cumulative dose, and primary treatment indication, and explored whether either regimen was associated with an early reduction in oral prednisone requirements. This study was designed to generate prospective comparative data on short-term clinical outcomes under two IVIG dosing strategies and to inform future dose-optimization studies.

## Methods

Patients with generalized myasthenia gravis (gMG) diagnosed according to accepted international criteria were prospectively recruited from the Department of Neuromuscular Diseases, The First Affiliated Hospital of Guangzhou University of Chinese Medicine (7, 8). All participants had not received human immunoglobulin therapy within 12 weeks before enrollment. Patients received either a low-dose IVIG regimen (target ≤0.2 g/kg/day) or a standard-dose regimen (target 0.4 g/kg/day) according to routine clinical practice and treating-physician judgment.

For the index IVIG course, we recorded the actual gram amount administered, number of infusion days, and primary clinical indication. Because the revision supplement sheet recorded gram amounts rather than weight-adjusted doses, daily dose (g/kg/day) and cumulative dose (g/kg) were calculated using baseline body weight from the primary dataset. Primary indications were categorized as acute exacerbation, crisis treatment, maintenance therapy, or perioperative/thymoma-related treatment.

Disease severity was assessed prospectively using the Myasthenia Gravis Activities of Daily Living (MG-ADL) scale and the Quantitative Myasthenia Gravis (QMG) score at baseline and at weeks 1, 2, 4, 8, and 12 after IVIG administration. Oral prednisone dose was recorded at baseline and week 12 to explore a short-term steroid-sparing signal.

## Statistics

Continuous variables are presented as median and interquartile range (IQR) or as mean with 95% confidence intervals (CI), and categorical variables as counts and percentages. Baseline group comparisons were performed using independent-samples t tests or Mann–Whitney U tests for continuous variables and chi-square tests or Fisher’s exact tests for categorical variables, as appropriate. Longitudinal MG-ADL and QMG results are presented descriptively by scheduled visit, and adjusted between-group comparisons at each visit were explored using regression models including age and thymoma status, which were selected *a priori* because of baseline imbalance. Prednisone dose change from baseline to week 12 was analyzed within groups using a paired t test or Wilcoxon signed-rank test according to the distribution of paired differences and between groups using an independent-samples t test, Welch’s t test, or Mann–Whitney U test as appropriate. Because this was an exploratory observational study, *p* values were not adjusted for multiple comparisons. Statistical analyses were performed using R, Microsoft Excel (Microsoft, Redmond, WA, United States, version 16.65), and IBM SPSS Statistics (IBM, Armonk, NY, USA, version 28.0.0.0). The study followed the Strengthening the Reporting of Observational Studies in Epidemiology (STROBE) reporting guidelines.

## Results

Thirty-four patients were included: 21 received low-dose IVIG (≤0.2 g/kg per day) and 13 received standard-dose IVIG (0.4 g/kg per day). At baseline, disease severity was similar between groups: median MG-ADL was 8 (IQR 4.5–13.0) vs. 6 (4.0–11.0) (*p* = 0.716), and median QMG was 18.0 (11.75–20.50) vs. 21.0 (12.50–25.75) (*p* = 0.391) in the low-dose and standard-dose groups, respectively. Groups were also comparable for sex (female 66.7% vs. 53.8%; *p* = 0.701), body weight (median 60.0 kg vs. 62.0 kg; *p* = 0.876), disease duration (median 7.0 years vs. 5.0 years; *p* = 0.390), and history of myasthenic crisis (47.6% vs. 53.8%; *p* = 1.000).

Baseline treatment patterns were broadly similar: any steroid use (28.6% vs. 15.4%; *p* = 0.444), any immunosuppressive therapy (0% vs. 7.7%; *p* = 0.382), concomitant steroid plus any immunosuppressive therapy (33.3% vs. 61.5%; *p* = 0.210), and no steroid and no immunosuppressive therapy (38.1% vs. 15.4%; *p* = 0.251). Refractory MG at baseline was uncommon and balanced (9.5% vs. 15.4%; *p* = 1.000). Previous thymectomy status categories were also similar across groups (not applicable 61.9% vs. 46.2% [*p* = 0.587]; within 1 year 9.5% vs. 7.7% [*p* = 1.000]; 1–5 years 9.5% vs. 23.1% [*p* = 0.348]; >5 years 19.0% vs. 23.1% [*p* = 1.000]).

Two clinically relevant imbalances were noted. First, patients in the standard-dose group were older (median 57 vs. 44 years; Table 1). Second, thymoma was more frequent in the standard-dose group (53.8% vs. 14.3%; *p* = 0.038). The overall distribution of MGFA classes differed (global *p* = 0.036), principally driven by more MGFA class V in the standard-dose cohort (23.1% vs. 0%; *p* = 0.048). To address these imbalances, age and thymoma status were prespecified covariates in adjusted analyses. Treatment exposure and primary indications for the index IVIG course are summarized in Supplementary Figure 1K. Most patients in both groups received 5 infusion days; in the low-dose cohort, one patient received 3 days and one received 6 days, whereas all patients in the standard-dose cohort received 5 days. After weight-based conversion, the low-dose group clustered around a daily exposure of approximately 0.2 g/kg/day and a cumulative exposure near 1.0 g/kg, whereas the standard-dose group clustered around approximately 0.4 g/kg/day and a cumulative exposure near 2.0 g/kg, with limited overlap between groups. Acute exacerbation was the most common indication in both cohorts (15/21 [71%] in the low-dose group and 5/13 [38%] in the standard-dose group). Crisis treatment accounted for 5/21 (24%) and 4/13 (31%) of courses, respectively. Maintenance therapy was observed only in the standard-dose cohort (4/13 [31%]), whereas perioperative/thymoma-related use was observed only in the low-dose cohort (1/21 [5%]).

**Table 1:** Baseline demographic and clinical characteristics.

Characteristic	Low-dose (*n* = 21)	Normal-dose (*n* = 13)	*p* value
Age (y), median (IQR)	44 (34.5,57.5)	57 (38.5,70.0)	0.473
Early-onset myasthenia gravis, *n*(%)	16 (76.2)	8 (61.5)	0.600
Sex, *n*(%)			0.701
Female	14 (66.7)	7 (53.8)	
Male	7 (33.3)	6 (46.2)	
Weight (kg), median (IQR)	60.00 (55.00,64.00)	62.00 (54.00,69.00)	0.876
Disease duration (y), median (IQR)	7.00 (2.50,12.5)	5.00 (3.00,13.00)	0.390
MGFA class at screening, *n*(%)			0.036
I	0 (0)	1 (7.7)	
IIa	3 (14.3)	5 (38.5)	
IIb	9 (42.9)	2 (15.4)	
IIIa	3 (14.3)	0 (0)	
IIIb	5 (23.8)	1 (7.7)	
IVb	1 (4.8)	1 (7.7)	
V	0 (0)	3 (23.1)	
Previous thymectomy, *n*(%)			0.693
Not applicable	13 (61.9)	6 (46.2)	0.587
Within 1 year after surgery	2 (9.5)	1 (7.7)	1.000
1–5 years after surgery	2 (9.5)	3 (23.1)	0.348
>5 years after surgery	4 (19.0)	3 (23.1)	1.000
Thymoma, *n*(%)	3 (14.3)	7 (53.8)	0.038
*n* missing	0	0	
History of myasthenic crisis, *n*(%)	10 (47.6)	7 (53.8)	1.000
Total MG-ADL score, median (IQR)	8.00 (4.50,13.00)	6 (4.00,11.00)	0.716
*n* missing	0	0	
Total QMG score, median (IQR)	18.00 (11.75,20.50)	21.00 (12.50,25.75)	0.391
*n* missing	0	0	
Myasthenia gravis therapy at baseline, *n*(%)			0.167
Any steroid	6 (28.6)	2 (15.4)	0.444
Any immunosuppressive therapy	0 (0)	1 (7.7)	0.382
Steroid and any immunosuppressive therapy	7 (33.3)	8 (61.5)	0.210
No steroid and no immunosuppressive therapy	8 (38.1)	2 (15.4)	0.251
n missing	0	0	
Refractory MG at baseline, *n*(%)	2 (9.5)	2 (15.4)	1.000
*n* missing	0	0	

AChR = acetylcholine receptor; IQR = interquartile range; IST = immunosuppressive therapy; MG-ADL = MG specific activity of daily life score; MGFA = Myasthenia Gravis Foundation of America classification; MG-QoL15 = MG-Specific Quality of Life Score; QMG = Quantitative MG Score. Reported are median (IQR) for continuous variables and *n* (%) for categorical variables. Disease duration is the time from diagnosis until baseline. The data on the thymoma findings refer to the number of patients in the respective group, regardless of whether a thymectomy was performed. We cannot exclude the possibility that a thymoma was present in the non-thymectomized patients, but there was no evidence of a thymoma on the CT scan of the thorax. A immunotherapy refractory status was defined according to the randomized controlled phase 3 trial of eculizumab in refractory, AChR-Ab-positive, generalized MG (REGAIN): diagnosis of a generalized AChR-Ab–positive MG with a MG-ADL score at baseline of >5 and a history of either 1 2 or more immunosuppressive therapies, or 2 at least one immunosuppressive therapy with IVIg or plasma exchange given at least 4 times per year, for 12 months without symptom control. *p* values were calculated using chi-square or Fisher’s exact tests for categorical variables and independent two-sample t-tests for continuous variables; the “n missing” row indicates the number of missing values, and *p* values are not reported for these rows.

Both regimens produced clinically meaningful improvements over 12 weeks. Observed longitudinal trajectories are shown in Supplementary Figure 1J. In both groups, MG-ADL and QMG scores declined from baseline over follow-up, and no statistically significant between-group differences were detected at any scheduled assessment (all *p* > 0.05). The standard-dose group showed greater variability at several intermediate visits, particularly at weeks 4 and 8, but week-12 mean scores were similar between groups. At week 12, response distributions were broadly comparable across groups (Supplementary Figures 1C,D for minimally improved MG-ADL and Supplementary Figures 1E,F for minimally improved QMG), and no between-group difference reached statistical significance.

Among patients receiving oral prednisone (low-dose *n* = 14; standard-dose *n* = 8), median daily doses did not decrease during follow-up (Table 2). In the low-dose cohort, doses were 11.25 mg (IQR 10.00–23.75) at baseline and 20.00 mg (7.50–23.75) at week 12 (within-group *p* = 0.653). In the standard-dose cohort, doses were 10.00 mg (10.00–20.00) at baseline and 20.00 mg (8.75–30.00) at week 12 (*p* = 0.388). No between-group differences were significant (all *p* > 0.05), and no clear short-term steroid-sparing signal was observed over 12 weeks.

**Table 2:** Oral prednisone dose at baseline and week 12 among patients receiving oral prednisone.

Group	*N*	Baseline dose	Follow-up dose	Δ change	% reduction	Within-group *p*
Low-dose	14	11.25 [10.00, 23.75]	20.00 [7.50, 23.75]	0.00 [−8.75, 6.88]	0.00 [−51.25, 47.50]	0.6530 (paired t)
Normal-dose	8	10.00 [10.00, 20.00]	20.00 [8.75, 30.00]	0.00 [−1.25, 10.00]	0.00 [−62.50, 6.25]	0.3884 (paired t)
Between-group comparison		0.5409 (Mann–Whitney)	0.8080 (indep t)	0.3831 (indep t)	0.4836 (indep t)	

## Discussion

In this prospective single-center cohort, both low-dose and standard-dose IVIG were associated with clinically meaningful improvement in MG-ADL and QMG over 12 weeks. Importantly, we did not detect statistically significant between-group differences at any scheduled follow-up visit, and the observed magnitude of improvement in the low-dose cohort was clinically comparable to that seen with standard dosing. At the same time, oral prednisone requirements did not decline over 12 weeks in either group, arguing against a clear short-term steroid-sparing signal. These findings support the possibility that a lower IVIG exposure may provide similar short-term symptom control in selected patients, but they should not be interpreted as definitive comparative evidence because of the observational design and limited sample size.

Current guideline recommendations generally use total IVIG doses of approximately 2 g/kg for acute worsening of MG, yet these schedules are supported more by accumulated practice than by dedicated dose-ranging trials (7–10). The supplementary treatment-exposure data in our cohort provide useful clinical context. Most patients in both groups received 5 infusion days, and after weight-based conversion the low-dose cohort clustered around a cumulative exposure of approximately 1.0 g/kg, whereas the standard-dose cohort clustered near 2.0 g/kg. Acute exacerbation was the most common indication in both groups, although the standard-dose cohort also included a substantial proportion of maintenance therapy courses, while the low-dose cohort included a small perioperative/thymoma-related subgroup. Recent randomized evidence also supports the clinical activity of maintenance IVIG in AChR-positive generalized MG, which provides useful context for the maintenance-treatment subgroup observed in our standard-dose cohort (11). These descriptive differences highlight that IVIG dose selection in routine practice is influenced not only by disease severity but also by treatment context, reinforcing the need for cautious interpretation in a non-randomized cohort.

The potential economic relevance of dose optimization remains important, but it should be framed cautiously. IVIG is costly and supply constraints are common, and MG-specific cost studies also suggest a substantial treatment burden associated with IVIG use; therefore, any strategy that preserves clinical benefit with lower cumulative exposure could have practical value (6, 12). However, our study did not collect formal cost, infusion-resource, hospitalization, or productivity data; therefore, no direct health-economic conclusion can be drawn from the present dataset. Instead, the current findings should be viewed as providing a clinical rationale for future prospective pharmacoeconomic evaluation.

The absence of a clear steroid-sparing signal underscores that the principal role of IVIG in this setting remains short-term symptom control rather than rapid corticosteroid tapering. Current guidance supports IVIG mainly for acute worsening and as a bridging treatment while slower-acting immunotherapies take effect (7, 8, 10). In our cohort, prednisone doses remained stable or increased slightly over follow-up regardless of IVIG regimen, suggesting that clinicians should not expect early steroid reduction within a 12-week window. This observation is also consistent with the possibility that corticosteroid adjustment in routine practice depends on multiple clinical considerations beyond early symptomatic improvement alone.

Therapeutic options for generalised myasthenia gravis are evolving rapidly, and our results must be considered alongside advances in complement inhibition and neonatal Fc-receptor (FcRn) blockade. Complement inhibitors such as eculizumab and ravulizumab neutralise the terminal complement cascade and have been shown to produce rapid and sustained improvements in MG with infrequent dosing (11, 13, 14). In the open-label extension of the CHAMPION MG trial, ravulizumab administered every eight weeks reduced rates of clinical deterioration and maintained improvements in MG-ADL and QMG scores over time (15), and a real-world study found that the largest improvements occurred within two weeks of initiation with responses stabilising thereafter (16). FcRn inhibitors like efgartigimod accelerate degradation of pathogenic IgG and thereby deplete acetylcholine receptor antibodies; in the ADAPT+ extension study, clinically meaningful reductions in MG-ADL and QMG scores were observed as early as one week and most participants achieved at least a three-point QMG improvement (17, 18). While these biologicals offer rapid, durable benefit, their high costs and uncertain long-term cost-effectiveness mean that optimising IVIG dosing remains an important consideration.

Several limitations deserve emphasis. First, this was a small, single-center, non-randomized cohort, and residual confounding cannot be excluded. Baseline imbalances in age, thymoma prevalence, and MGFA class may have influenced both treatment selection and outcome trajectories. Second, although earlier drafts used stronger comparative efficacy language, the present study did not prespecify a formal decision margin or a corresponding *a priori* power calculation; accordingly, the results are more appropriately interpreted as showing no statistically significant between-group difference rather than establishing definitive comparative effectiveness. Third, the follow-up period was limited to 12 weeks and may have been too short to capture delayed steroid-sparing effects or longer-term differences in disease control. Finally, treatment indication and dosing schedule varied across patients, reflecting real-world practice but also introducing additional clinical heterogeneity.

Future research should build upon these findings. A multicentre, randomised controlled trial comparing low-dose and standard-dose IVIG could more definitively evaluate the relative clinical effectiveness of these dosing strategies, as well as long-term efficacy, steroid-sparing potential, and health-economic outcomes. Incorporating pharmacokinetic measurements such as serum IgG levels, FcRn occupancy and antibody titres may help tailor dosing to individual patients. Economic analyses should quantify whether reduced daily dosing translates into lower direct medical costs, shorter infusion times and reduced burden on healthcare resources. Comparative studies with complement inhibitors and FcRn antagonists will clarify the relative benefits, risks and costs of IVIG versus targeted biologics, and may reveal additive or synergistic effects. Evaluating subcutaneous immunoglobulin, which provides more stable IgG levels and greater patient autonomy, is also important (8). Ultimately, integrating low-dose IVIG into a precision medicine framework alongside emerging biologics and cost-effectiveness considerations may yield more effective, sustainable treatment strategies for myasthenia gravis.

## Data Availability

The data analyzed in this study is subject to the following licenses/restrictions: the dataset contains patient-level clinical information and is therefore restricted to protect participant privacy and comply with institutional ethics/data-protection requirements. De-identified data may be made available from the corresponding author upon reasonable request, subject to approval by the relevant ethics committee/institution and any required data-use agreement. Requests to access these datasets should be directed to 1922953732@qq.com.
