# Supplementary figures and images for: Low-dose versus standard-dose intravenous immunoglobulin in generalized myasthenia gravis: a prospective single-center cohort study
Source: Front Neurol. 2026 Apr 28;17:1780647. doi: 10.3389/fneur.2026.1780647 (PMC13160847; doi:10.3389/fneur.2026.1780647)

(C) (D)

(E) (F)


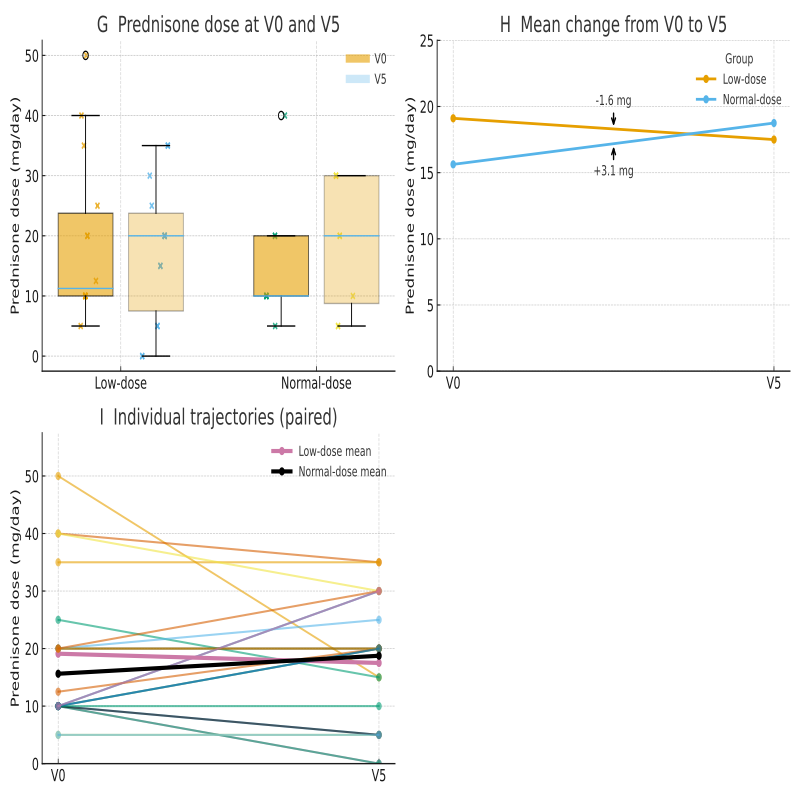


(J)


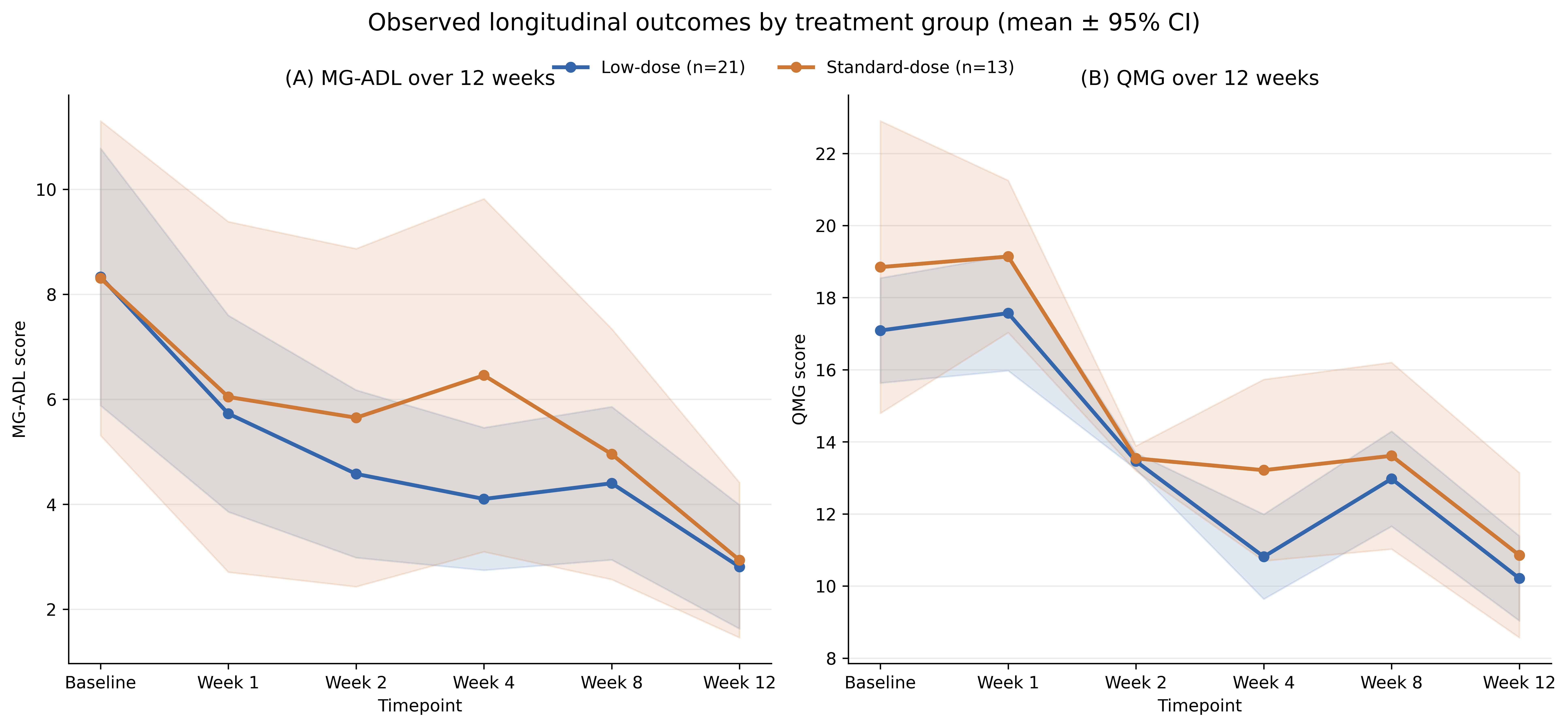


(K)


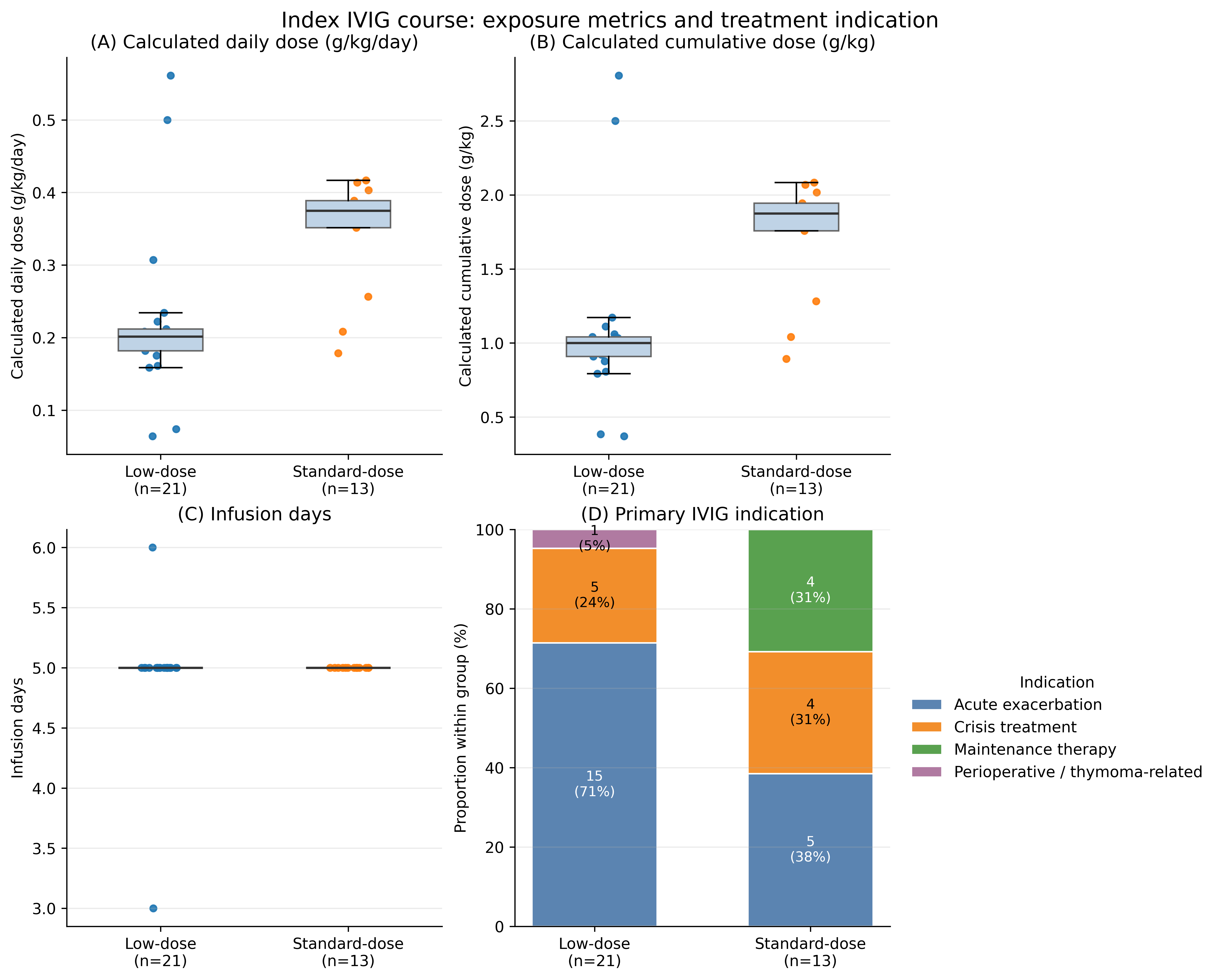

Supplement: Supplementary file 1 [file Data_Sheet_1.docx]
